# Evolutionary Game Analysis of Farmers' Conservation Tillage Behavior in Black Soil Areas Guided by Deep Learning

**DOI:** 10.1155/2022/5999007

**Published:** 2022-06-25

**Authors:** Na Meng, Jing Zhou

**Affiliations:** School of Economics and Management, Shenyang Agricultural University, Shenyang 110866, China

## Abstract

To better protect the rights and interests of farmers, the evolutionary game theory and deep learning (DL) technology are used to analyze the conservation tillage behavior of farmers in black soil areas. Firstly, the basic hypotheses are put forward and an evolutionary game model is constructed. Secondly, the evolutionary game model between farmers and the government is analyzed, and dynamic equations are built. Finally, the model is deduced and studied, and a Convolutional Neural Network (CNN) with a double-sized convolution kernel is constructed to classify the land of remote sensing images. The experimental results manifest that after the dynamic evolutionary game, the net income generated by farmers adopting the conservation tillage strategy without government regulation is positive. The net income of government regulation is positive, and the game equilibrium point is (1, 0). After a dynamic evolutionary game, the game is balanced when the government does not regulate, the net income generated by farmers adopting conservation tillage strategies and the net income generated by government regulation and farmers adopting conservation tillage strategies are negative, the net income from government regulation is positive, and the game equilibrium point is (0, 1). The constructed CNN can achieve 91.32% overall accuracy for black soil classification, and the proposed scheme provides some references for the application of CNN in evolutionary games.

## 1. Introduction

Game theory refers to changing one's own strategy according to the strategy of others to achieve the goal of winning. The idea of game theory has existed since ancient times. The famous ancient military book *The Art of War* is the earliest work involving game theory [1, 2]. Game theory was originally designed to study the problem of winning and losing in gambling, but people's guesses about the outcome of gambling are only based on experience and have not been promoted to theory. Modern research on game theory began with Zermelo, Borel, and John von Neumann. Game theory studies the behavior of individuals in the game, including the actions they intend to take and actually take [3, 4], and studies the optimization strategies of Chen et al. [5].

It continues to study the foundations and deficiencies of previous work. The evolutionary game model for farmers and the government to choose the “smart agriculture” mode is established, and the evolutionary path and evolutionary equilibrium are analyzed. On this basis, countermeasures and suggestions are given, and the purpose is to help the evolution proceed in a mutually beneficial direction. There are two innovations in the research. The first innovation is to establish an evolutionary game model for farmers and the government to choose the “smart agriculture” mode. The second innovation is to introduce the deep learning (DL) technology into the analysis of farmers' conservation tillage behavior.  Section 1 describes the research background.  Section 2 analyzes and describes the theoretical mechanism of evolutionary games.  Section 3 analyzes and constructs the game payoff matrix.  Section 4 analyzes the experimental results of the payoff matrix, and Section 5 summarizes the research content and proposes the future research direction.

## 2. Literature Review

Researchers have carried out a lot of research work in the corresponding fields. Abadi et al. (2021) [6] proposed the factors predicting the behavioral intentions of medicinal and aromatic plants in farmland by comparing the psychosocial and microeconomic characteristics of adopters and non-adopters. In contrast to previous studies establishing inductive hypotheses, the present study in turn benefits from a deductive approach and recognizes knowledge gaps in attitude-intent behavior and cost-benefit behavior in the context of aromatic plant cultivation. In conclusion, the findings provide insights for agricultural decision-makers and extension agencies in promoting the cultivation of aromatic plants as a complement to traditional cultivation patterns. Qiu et al. (2021) [7] explored the interaction between farmers' happiness and ecological protection cognition, attitude, and behavior. The data used in the research were 618 survey data in the Qinling area in 2018, and a structural equation model was constructed. Theoretical hypotheses on the interaction of farmers' happiness, conservation cognition, conservation attitudes, and conservation behaviors were proposed and validated. The results showed that the happiness effect of farmers with a positive attitude towards protection was generally stronger, and farmers with higher happiness had an incentive effect on their ecological protection behavior. Li et al. (2021) [8] analyzed the key factors affecting farmers' willingness, behavior, and consistency of willingness and behavior to adopt photovoltaic agriculture. The results demonstrated that 37.1% of the farmers' adoption intentions were consistent with their adoption behaviors, while 62.9% of the farmers' adoption intentions were inconsistent with their adoption behaviors. There are differences in the key factors affecting the willingness, behavior, and consistency of willingness and behavior of photovoltaic agriculture adoption. Lee et al. (2021) [9] pointed out that the price of agricultural products will have a lot of fluctuations over time. Traditional time series and stochastic forecasting methods may not capture this dynamic. They tried to use machine learning to adjust the model to the actual situation by building the price determination mechanism on stochastic automata and evolutionary game models. The experimental results denoted that no matter what the initial distribution of crops is, the spatial aggregation distribution leads to the greater fluctuation of the time series. Wang et al. (2021) [10] proposed that with the continuous development of the evolutionary game theory, evolutionary games are widely used in various fields of society. An evolutionary game model of forest resource management actors and governance systems is constructed, and the stability strategies of each break-even point of the game system are analyzed. Nicolas et al. [11] used system dynamics, the evolutionary game theory, and the principal-agent theory to analyze the principal-agent problem between business owners and professional managers, and constructed a principal-agent evolutionary game model. They also analyzed the law of strategic choice and predicted the equilibrium results under different scenarios. The research results indicated that the basic benefits and costs of cooperation are the key factors of strategic choice, and the difference of the expected benefits of different strategies will also affect the selection probability of these cooperation strategies. By combing various relevant researches, it can be seen that domestic and foreign scholars have achieved quite rich results in the theoretical and empirical research on farmers' technology selection behavior and conservation tillage adoption behavior. The results of our predecessors provide a good theoretical basis and reference for this research and have important implications for this research. Throughout the relevant researches around the world, the specific factors affecting farmers' technology choice behavior and conservation tillage adoption behavior can be summarized into three categories: First, the individual characteristics of the farmers, including age, education level, technical training, information acquisition and knowledge of technology, etc. Second, the family characteristics of the farmers, including the scale of operation, the number of family labor, and the family's economic status. Third, external factors, including the policy, environment, and farmland endowments. In the actual technology selection process of farmers, these factors are more likely to act at the same time, but there are differences in the magnitude and direction of the effects. In addition, the application benefits of the conservation tillage technology can basically be summarized as economic benefits, social benefits, and ecological benefits. The application of the conservation tillage technology in developed countries developed rapidly. However, the promotion of the conservation tillage technology in China is relatively lagging behind. As an upgrade of the traditional farming model, conservation tillage is an ecological and environment-friendly farming mode, which can be regarded as an important means to promote the sustainable development of modern agriculture in China. In general, there is still a lot of room for research on the promotion and application of the conservation tillage technology in China.

## 3. Evolutionary Game and the Construction of the DL Model

### 3.1. Farmer's Behavior Theory and Evolutionary Game

There are three main schools of research on contemporary farmers' behavior, namely rational small farmers school, organization school, and bounded rationality hypothesis. The school of rational small farmers is represented by the American economist Theodore W. Schultz, whose masterpiece “Transforming Traditional Agriculture” mainly discusses the rational behavior of farmers. He considers that in traditional agriculture, under specific production conditions and resource constraints, farmers have completely rational behaviors in pursuit of profit maximization and cost minimization. Therefore, the main reason for the decline of traditional agriculture is the diminishing marginal returns of traditional agriculture, rather than changes in the farmers' production behavior and market competitiveness [12, 13]. Underdeveloped countries are eager to develop their economy, and hence they attach great importance to the development of industry, but neglect the improvement of agricultural production technology, resulting in the backwardness of the agricultural development level. The increase in agricultural production is mainly due to the increase of various production factors, and it is difficult to promote the development of agriculture only by readjusting the agricultural production factors. The organization school, represented by the former Soviet Union economist A. Chayanov, analyzed the difference between the capitalist economy and the small-scale peasant family economy and concluded that the development of the small-scale peasant family economy cannot use the theory of capitalist economics. He believes that the determinants of the normal functioning of small-scale peasant family farms are labor supply and satisfactory consumption. The bounded rationality hypothesis is represented by Simon, who believes that the “economic man” theory that pursues the maximization of interests is not in line with reality because this theory assumes that people are completely rational. He thinks that the information people receive is not complete, the external environment is uncertain, people's computing power is limited, etc. These factors make people's rationality limited. Due to the bounded rationality of human beings, people mainly follow the principle of satisfaction when making decisions [14–16].

The evolutionary game theory originated from scholars' research on conflicts and cooperation between animals and plants in biology. They found that the game results between animals and plants are based on imperfect rationality. Animals and plants also imitate and learn from each other, seeking the best cooperation opportunities for both parties, but evolutionary games were not formally proposed at that time. The evolutionary game theory is based on game theory. It is an extension and development of game theory and a process of dynamic development of game theory. The evolutionary game theory has two important concepts, namely the evolutionary stability strategy and the replicator dynamic equation. The evolutionary stability strategy is the final game result of both sides of the game, and it is a long-term steady-state process, while the replicator dynamic equation represents the process of the steady-state approach of the game direction, which is a time function. Because of the limited degree of rationality of human beings, the choices between the game parties are usually variable, and only after repeated choices by both parties can a satisfactory result be obtained [17–19].

### 3.2. DL Theory and Model Construction

**Figure 1:**
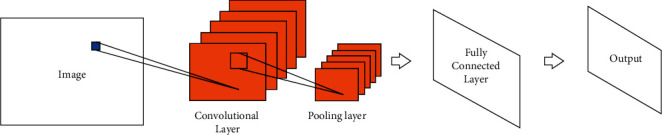
The structure of the CNN.

**Figure 2:**
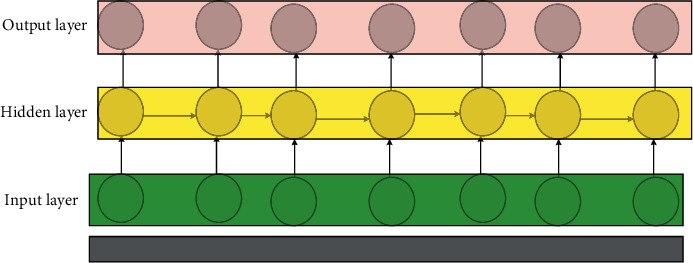
The structure of the RNN.

DL is one of the main paths to artificial intelligence (AI). It is a branch of machine learning that essentially fits data to summarize available patterns. DL has achieved great success, greatly promoted the development of technology, and produced a profound impact. The Convolutional Neural Network (CNN) is one of the most important models in DL. Since 2012, it has been the most important model of DL in the field of image processing. Combining with other techniques enables convolutional neural networks to be applied in many different fields. The first true CNN was LeNet proposed in 1998. The network has been widely used in the recognition of handwritten fonts of the Bank of America checks and achieved good results. The Recurrent Neural Network (RNN) is another commonly used type of network in the field of DL. It has a memory function, is suitable for solving continuous sequence problems, and is good at learning rules from samples with certain sequential meanings [20, 21], unlike the CNN, which is generally used to deal with image problems. The structure of the CNN is shown in Figure 1. The RNN is generally used in fields such as production and prediction. The structure of the RNN is shown in Figure 2.

Compared with the fully connected neural network, the RNN increases the self-circulation of hidden neurons and the interconnection between hidden neurons in the hidden layer. Since the input of the RNN is time-series information, and the state of neurons is continuously updated through iterative operations, the hidden layer in the RNN has timing. In the RNN, because the data it processes have a strong logical relationship, for each step of the input of the hidden layer, all operations share the weight coefficient parameters. In this way, the network only needs to train a set of weight coefficient matrices. There is no need to adjust the weight coefficient matrix according to the input length, which reduces the number of parameters that need to be learned in the network. The RNN is a model for processing time-series information. The input received at each moment is a feature vector. During the entire input process of the network, the vectors at different times are input to the network in sequence, and this string of vectors can be regarded as a matrix. The output of the network is also a vector.

**Figure 3:**
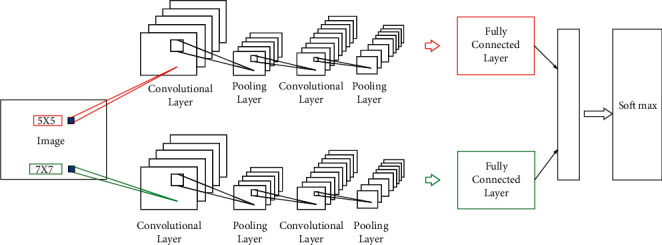
The constructed CNN.

The research uses CNNs to extract deeper and more complex spectral features from remote sensing images as the basis to extract black soil information for classification. The CNN is trained to extract the desired features. The CNN outputs the feature map of the input image. The design of the first convolutional layer is particularly important. If the convolution kernel of the first layer of convolution is too large, some details of the image will be lost. If the convolution kernel is too small, the features of the image cannot be presented [22, 23]. Traditional CNNs use fixed-size convolution kernels, and thus image granularity is also fixed. In this way, some features will be lost in the process of learning, thus reducing the accuracy of network recognition [24–27]. Therefore, a convolutional neural network with double-sized convolution kernels has two convolutional layers, two pooling layers, and one fully connected layer. The convolution kernel sizes of the first convolutional layers of the two CNNs are different, which are 5*∗*5 and 7*∗*7, respectively, as shown in Figure 3. The fully connected layers of the two CNNs are combined using a fully connected map, and finally input into the classifier for feature classification. The constructed CNN is shown in Figure 3.

## 4. Analysis of Game Evolution and Stability Strategy between Farmers and the Government

### 4.1. Establishment of the Model

Assume two-game groups: the government and farmers. When the government requires farmers to implement the conservation tillage strategy, farmers can choose to implement the strategy to protect the soil quality of the black soil area and optimize the ecological environment. They can also choose not to implement the strategy. The government can choose to regulate farmers' choice of conservation tillage strategies, such as introducing conservation tillage supervision policies, providing policy subsidies, and imposing economic sanctions (fines, taxes) on violations; or they can choose to not regulate. As a result, the game payoff matrix between the government and farmers is established as shown in Table 1.

The meanings of the parameters are explained as follows: *C*_*f*_ represents the implementation cost of farmers adopting the conservation tillage strategy; *C*g means the cost when the government implements the environmental regulation strategy; *R*_*i*_ refers to the economic benefits obtained by farmers when they adopt the conservation tillage strategy; *R*_*e*_ shows that when farmers adopt conservation tillage strategies, the environmental benefits brought about by changes in soil quality of black soil; *L* indicates the loss of soil degradation in black soil when farmers do not adopt conservation tillage strategies; *F* expresses that farmers are fined by the government when they do not adopt the conservation tillage strategy; *λ* denotes the weight coefficient of the economic level of farmers in the total economic level of farmers in the country, 0 < *λ* < 1; *μ* manifests the weight coefficient of the farmland quality of farmers in the total quality of the cultivated land in the black soil areas of the country, 0 < *β* < 1; *α* demonstrates the coefficient of the degree of farmers adopting conservation tillage strategies; the greater the coefficient, the greater the degree of farmers adopting conservation tillage techniques, 0 ≤ *α* ≤ 1; *β* stands for the coefficient of the degree of environmental regulation implemented by the government. The larger the coefficient, the stricter the degree of environmental regulation, 0 < *β* < 1 [28, 29].

### 4.2. Construction of Dynamic Equations

In the initial stage, it is assumed that the probability of farmers implementing the conservation tillage strategy is *x*(0⩽*x*⩽1), and the probability of not adopting conservation tillage is 1 − x. The probability of the government choosing a regulatory policy is *y*(0⩽*y*⩽1), and the probability of non-regulation is 1 − *y*. The expected benefit and the group benefit of farmers adopting and not adopting the conservation tillage strategy are calculated as *U*_1_, *U*_2_, and ${\bar{U}}_{12}$, respectively, and their mathematical expressions are shown in equations (1)–(3):

(1) $$\begin{array}{c} U_{1}=\;y\left(-C_{f}+R_{i}+R_{e}\right)+\left(1-y\right)\left(-C_{f}+R_{i}+R_{e}\right), \end{array}$$

(2) $$\begin{array}{c} U_{2}=\;y\left(-\alpha C_{f}-F-\alpha L\right)+\left(1-y\right)\left(-\alpha C_{f}-\beta F-\alpha L\right), \end{array}$$

(3) $$\begin{array}{c} {\bar{U}}_{12}=\;xU_{1}+\left(1-x\right)U_{2}. \end{array}$$

*C*_*f*_ represents the implementation cost of farmers adopting the conservation tillage strategy; *R*_*i*_ refers to the economic benefits obtained by farmers when they adopt the conservation tillage strategy; *R*_*e*_ shows that when farmers adopt conservation tillage strategies, the environmental benefits brought about by changes in soil quality of black soil; *L* indicates the loss of soil degradation in black soil when farmers do not adopt conservation tillage strategies; *F* expresses that farmers are fined by the government when they do not adopt the conservation tillage strategy; *β* manifests the weight coefficient of the farmland quality of farmers in the total quality of the cultivated land in the black soil areas of the country, 0 < *β* < 1; *α* demonstrates the coefficient of the degree of farmers adopting conservation tillage strategies; the greater the coefficient, the greater the degree of farmers adopting conservation tillage techniques, 0 ≤ *α* ≤ 1.

According to the Malthusian dynamic equation, when the return of a certain strategy selected in the game problem is higher than the average return of other strategies of the group, it is considered that the strategy can adapt to the evolution process of the group and has strong resistance to prevent the invasion of mutation strategies. Then, the replicator dynamics equation of farmers adopting the conservation tillage strategy is shown in equation (4):

(4) $$\begin{array}{c} \frac{\text{d}x}{\text{d}t}=x\left(U_{1}-{\bar{U}}_{12}\right)=\;x\left[U_{1}-xU_{1}-\left(1-x\right)U_{2}\right]=x\left(1-x\right)\left(U_{1}-U_{2}\right), \end{array}$$

*U*_1_ and *U*_2_ are substituted into the replicator dynamics equation to obtain the mathematical relationship, as shown in equation (5):

(5) $$\begin{array}{c} \frac{\text{d}x}{\text{d}t}=x\left(1-x\right)\left[yF\left(1-\alpha \right)-C_{f}\left(1-\alpha \right)+\beta F+\alpha L-R_{i}-R_{e}\right]. \end{array}$$

In terms of government, the expected benefit and the group benefit of choosing to implement the regulation and not to implement the regulation strategy are *U*_3_, *U*_4_, and ${\bar{U}}_{34}$, respectively, as shown in equations (6)–(8):

(6) $$\begin{array}{c} U_{3}=\;x\left(-C_{g}+\lambda R_{i}+\mu R_{e}\right)+\left(1-x\right)\left(-C_{g}+F-\alpha \mu L\right), \end{array}$$

(7) $$\begin{array}{c} U_{4}=\;x+\left(1-x\right)\left(-\beta C_{g}+\beta F-\alpha \mu L\right), \end{array}$$

(8) $$\begin{array}{c} {\bar{U}}_{34}=\;yU_{3}{\left(1-y\right)}_{4}. \end{array}$$

The replicator dynamics equation of the government's choice of regulation strategy is shown in equation (9):

(9) $$\begin{array}{c} \frac{\text{d}y}{\text{d}t}=\;y\left(U_{3}-{\bar{U}}_{34}\right)=\;y\left[U_{3}-yU_{3}-\left(1-y\right)U_{4}\right]=y\left(1-y\right)\left(U_{3}-U_{4}\right). \end{array}$$

*U*_*3*_ and *U*_*4*_ are substituted into the replicator dynamics equation to obtain the mathematical relationship, as shown in equation (10):

(10) $$\begin{array}{c} \frac{\text{d}y}{\text{d}t}=y\left(1-y\right)\left[-C_{g}\left(1-\beta \right)-xF\left(1-\beta \right)+F\left(1-\beta \right)\right]. \end{array}$$

Combining equations (5) and (10), the replicator dynamics system of the government and farmers is obtained, as shown in equation (11):

(11) $$\begin{array}{c} \left\{\begin{array}{c} \frac{\text{d}x}{\text{d}t}=\;x\left(1-x\right)\left[yF\left(1-\beta \right)-\;C_{f}\left(1-\alpha \right)yF\left(1-\beta \right)-\;C_{f}\left(1-\alpha \right)\right.\;\left.+\beta F+\alpha L-R_{i}-R_{e}\right]\\ \frac{\text{d}y}{\text{d}t}=y\left(1-y\right)\left[-C_{g}\left(1-\beta \right)-xF\left(1-\beta \right)\right.\left.+\;F\left(1-\beta \right)\right] \end{array}\right. \end{array}$$

Through equation (11), it is obtained that there are 5 replicate dynamic equilibrium points on the plane *p*={(*x*, *y*)*|*0⩽*x*, *y*⩽1} for the game system between the government and farmers: (0, 0), (1, 0), (1, 1), (0, 1), and (−*C*_*g*_1 − *β*+*F*(1 − *β*)/*F*(1 − *β*)), (*C*_*f*_1 − *α* − *βF* − *αL*+*R*_*i*_+*R*_*e*_/*F*(1 − *β*)) (the postscript is  *x* ^*∗*^,  *y* ^*∗*^). If 0≤ −*C*_*g*_(1 − *β*) − *F*(1 − *β*)/*F*(1 − *β*) ≤1 and 0≤ *C*_*f*_1 − *α* − *βF* − *αL*+*R*_*i*_+*R*_*e*_/*F*(1 − *β*) ≤1, it is established.

The stability of the equilibrium point of the evolutionary system can be determined by the local asymptotic stability analysis method of the Jacobi matrix. Taking the partial derivatives with respect to the sum of equation (11), respectively, the system Jacobi matrix can be obtained, as shown in equation (12):

(12) $$\begin{array}{c} J=\left[\begin{array}{c} \left(1-2x\right)\left[yF\left(1|-|\beta \right)-\;C_{f}\left(1-\alpha \right)+\beta F+\alpha L\right.\\ \left.-R_{i}-R_{e}\right]\;F\left(1-x\right)\\ F\left(y-1\right)y\left(1-2y\right)\left[-C_{g}\left(1-\beta \right)-xF\left(1-\beta \right)\right.\\ \left.+\;F\left(1-\beta \right)\right] \end{array}\right] \end{array}$$

The determinant of matrix *J* is shown in equation (13):

(13) $$\begin{array}{c} \det J=1-2x\left[yF1-\beta -\;C_{f}1-\alpha +\beta F\;+\alpha L-R_{i}-R_{e}\right]1-2y\left[-C_{g}1-\beta -xF1-\beta +\;F1-\beta \right]-\;F\left(1-x\right)xF\left(y-1\right)y. \end{array}$$

The trace of matrix *J* is shown in equation (14):

(14) $$\begin{array}{c} trJ=1-2x\left[yF1-\beta -\;C_{f}1-\alpha +\beta F\;+\alpha L-R_{i}-R_{e}\right]+1-2y\left[-C_{g}1-\beta -xF1-\beta +\;F1-\beta \right]. \end{array}$$

When the Jacobi matrix determinant (det*J*) of the equilibrium point satisfies det*J* >0 and the trace (tr*J*) satisfies tr*J* < 0, it is a local asymptotically stable fixed point in the dynamic process of the system evolution, corresponding to the evolutionary stability strategy. Meanwhile, the evolutionary game stability strategy must have the ability to resist disturbance in the stable state, i.e., satisfy d*x*/d*t* < 0 and d*y*/d*t* > 0. Therefore, the stability of the evolution equilibrium point of the game system between the government and farmers is further analyzed according to the different value ranges of the parameters.

### 4.3. Derivation and Analysis of the Model

According to the value of different parameters, the equation results of det*J* and tr*J* are obtained, as shown in Table 2. When the government does not regulate, the net income generated by farmers in the black soil area adopting the conservation tillage strategy is expressed as −*C*_*f*_(1 − *α*)+*βF*+*αL* − *R*_*i*_ − *R*_*e*_＞0. When the government conducts regulation, the net income generated by farmers adopting the conservation tillage strategy in the black soil area is expressed as *F* − *C*_*f*_(1 − *α*)+*αL* − *R*_*i*_ − *R*_*e*_＞0. When the government implements the regulation strategy, the net benefit is (*F* − *C*_*g*_)(1 − *β*).

According to the different value ranges of the parameters, the equilibrium point of the replicator dynamics system is obtained from the equation group (11). The following six inferences can be obtained:

1. When −*C*_*f*_(1 − *α*)+*βF*+*αL* − *R*_*i*_ − *R*_*e*_＞0, (*F* − *C*_*g*_)(1 − *β*) > 0, four equilibrium points of the replicator dynamics system are obtained, namely (0, 0), (1, 0), (1, 1), and (0, 1). After the dynamic evolutionary game, the government does not regulate, the net income generated by farmers adopting the conservation tillage strategy and the net income of government regulation are all positive, and the game equilibrium point is (1, 0). It means that farmers will eventually choose to carry out conservation tillage behavior and the government will eventually choose to praise them.
2. When −*C*_*f*_(1 − *α*)+*βF*+*αL* − *R*_*i*_ − *R*_*e*_＞0, (*F* − *C*_*g*_)(1 − *β*)＜0, four equilibrium points of the replicator dynamics system are obtained, namely (0, 0), (1, 0), (1, 1), and (0, 1). After the dynamic evolutionary game, the net income generated by the government without regulation and farmers adopting the conservation tillage strategy is positive, the net income of government regulation is positive, and the game equilibrium point is (1, 0). It refers that farmers will eventually choose to carry out conservation tillage behavior and the government will eventually choose to praise them.
3. When −*C*_*f*_(1 − *α*)+*βF*+*αL* − *R*_*i*_ − *R*_*e*_＜0, *F* − *C*_*f*_(1 − *α*)+*αL* − *R*_*i*_ − *R*_*e*_＜0, and (*F* − *C*_*g*_)(1 − *β*)＜0, four equilibrium points of the replicator dynamics system are obtained, namely (0, 0), (1, 0), (1, 1), and (0, 1). After the dynamic evolutionary game, when the government does not conduct regulation and the net income generated by farmers adopting the conservation tillage strategy is negative, the net income generated by the government conducting regulation and farmers adopting the conservation tillage strategy and the net income from government regulation are both negative, and the game equilibrium point is (0, 0). It indicates that farmers will eventually choose not to carry out conservation tillage and the government will eventually choose not to punish, or farmers will eventually choose to carry out conservation tillage and the government will eventually choose to praise farmers; the final choice (no protection, punishment) or choice (protection, praise) depends entirely on the position of the point ( *x* ^*∗*^,  *y* ^*∗*^). When the position of the point ( *x* ^*∗*^,  *y* ^*∗*^)moves to the lower left, the more likely the initial point is to the upper right.
4. When −*C*_*f*_(1 − *α*)+*βF*+*αL* − *R*_*i*_ − *R*_*e*_＜0, *F* − *C*_*f*_(1 − *α*)+*αL* − *R*_*i*_ − *R*_*e*_＜0, and (*F* − *C*_*g*_)(1 − *β*)＞0, four equilibrium points of the replicator dynamics system are obtained, namely (0, 0), (1, 0), (1, 1), and (0, 1). After a dynamic evolutionary game, when the government does not regulate, the net income generated by farmers adopting the conservation tillage strategy and the net income generated by the government regulation and farmers adopting the conservation tillage strategy are negative, the net income from government regulation is positive, and the game equilibrium point is (0, 1). It demonstrates that farmers will eventually choose not to carry out conservation tillage behavior and the government will eventually choose to punish.
5. When −*C*_*f*_(1 − *α*)+*βF*+*αL* − *R*_*i*_ − *R*_*e*_＜0, *F* − *C*_*f*_(1 − *α*)+*αL* − *R*_*i*_ − *R*_*e*_＞0, and (*F* − *C*_*g*_)(1 − *β*)＜0, four equilibrium points of the replicator dynamics system are obtained, namely (0, 0), (1, 0), (1, 1), and (0, 1). After a dynamic evolutionary game, when the government does not regulate, the net income generated by farmers adopting the conservation tillage strategy and the net income generated by the government regulation and farmers adopting the conservation tillage strategy are negative, the net income from government regulation is positive, and the game equilibrium point is (0, 1). It denotes that farmers will eventually choose not to carry out conservation tillage behavior and the government will eventually choose to punish.
6. When −*C*_*f*_(1 − *α*)+*βF*+*αL* − *R*_*i*_ − *R*_*e*_＜0, *F* − *C*_*f*_(1 − *α*)+*αL* − *R*_*i*_ − *R*_*e*_＞0, and (*F* − *C*_*g*_)(1 − *β*)＞0, five equilibrium points of the replicator dynamics system are obtained, namely (0, 0), (1, 0), (1, 1), (0, 1), and ( *x* ^*∗*^,  *y* ^*∗*^). After the dynamic evolutionary game, when the government does not conduct regulation, the net income generated by farmers adopting the conservation tillage strategy is negative, and when the government conducts regulation, the net income generated by farmers adopting the conservation tillage strategy and the net income from government regulation are positive, there is no evolutionary stable strategy between the government and farmers, and the center point ( *x* ^*∗*^,  *y* ^*∗*^) is obtained, and both sides choose a mixed strategy.

For the above-mentioned six situations, refer to Table 3 for details:

## 5. Numerical Simulation Analysis of Evolutionary Game and Analysis of the Experiment Results of DL

### 5.1. Numerical Simulation Analysis of Evolutionary Game

**Figure 4:**
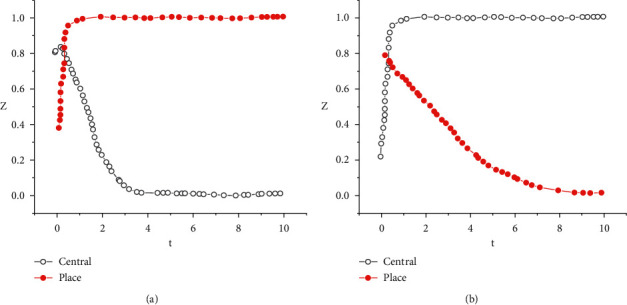
The dynamic evolution diagram of the evolutionary game model. (a) Government regulates, farmer does not adopt; (b). Government does not regulate, farmer adopts.

The dynamic evolution diagram of the evolutionary game model is shown in Figure 4.

Figure 4 denotes that when the government regulates and the farmer does not adopt conservation tillage, the value of *X* drops rapidly when the time is 0–2; when the time is greater than 2, the value of *X* remains stable. When the time is 0–2, the value of *Y* rises rapidly; when the time is greater than 2, the value of *Y* remains stable. When the government does not regulate and the farmer adopts conservation tillage, the changes in *X* and *Y* are equal.

**Figure 5:**
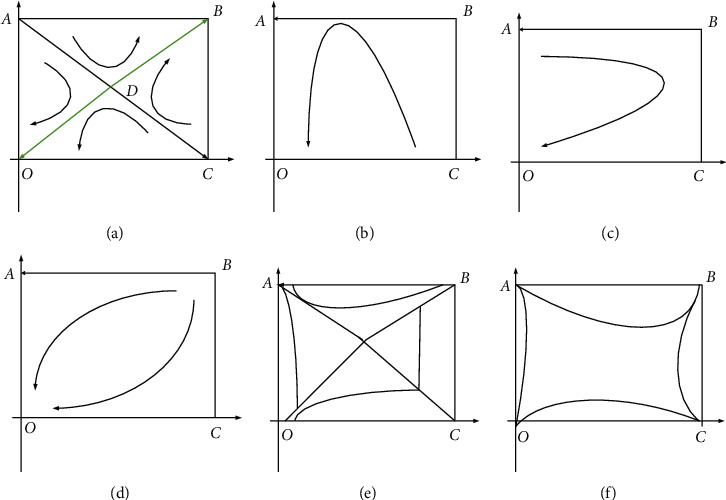
The evolution path diagram (a) Inference 1; (b) Inference 2; (c) Inference 3; (d) Inference 4; (e) Inference 5; and (f) Inference 6.

Figure 5 shows the evolution path diagram of the six inferences in Section 4.3.

In Figure 5, Figure 5(a) refers that the evolution result is uncertain in the case, and it is very meaningful to the analysis of the first case.  Figure 5(b) shows that the quadrilateral ABCO can be divided into two parts, and the upper right part is ABC. If the initial state falls in this part, the evolution result will be more inclined towards the equilibrium point B. That is to say, in this case, the government chooses the “conservation tillage” model, and farmers also agree to carry out this model.

**Inference 1 .**

After the model goes through a dynamic evolutionary game, the government does not regulate, the net income generated by farmers adopting the conservation tillage strategy and the net income of government regulation are all positive, and the game equilibrium point is (1, 0).

**Inference 2 .**

After the model goes through a dynamic evolutionary game, the net income generated by the government without regulation and farmers adopting the conservation tillage strategy is positive, the net income of government regulation is positive, and the game equilibrium point is (0, 1).

**Inference 3 .**

After the model goes through a dynamic evolutionary game, when the government does not conduct regulation and the net income generated by farmers adopting the conservation tillage strategy is negative, the net income generated by the government conducting regulation and farmers adopting the conservation tillage strategy and the net income from government regulation are both negative, and the game equilibrium point is (0, 0).

**Inference 4 .**

After the model goes through a dynamic evolutionary game, when the government does not regulate, the net income generated by farmers adopting the conservation tillage strategy and the net income generated by the government regulation and farmers adopting the conservation tillage strategy are negative, the net income from government regulation is positive, and the game equilibrium point is (0, 1).

**Inference 5 .**

After the model goes through a dynamic evolutionary game, when the government does not regulate, the net income generated by farmers adopting the conservation tillage strategy and the net income generated by the government regulation and farmers adopting the conservation tillage strategy are negative, the net income from government regulation is negative, and the game equilibrium point is (0, 0).

**Inference 6 .**

After the model goes through a dynamic evolutionary game, when the government does not conduct regulation, the net income generated by farmers adopting the conservation tillage strategy is negative, and when the government conducts regulation, the net income generated by farmers adopting the conservation tillage strategy and the net income from government regulation are positive, and there is no evolutionary stable strategy between the government and farmers.

### 5.2. Analysis of the Experimental Results of DL

**Figure 6:**
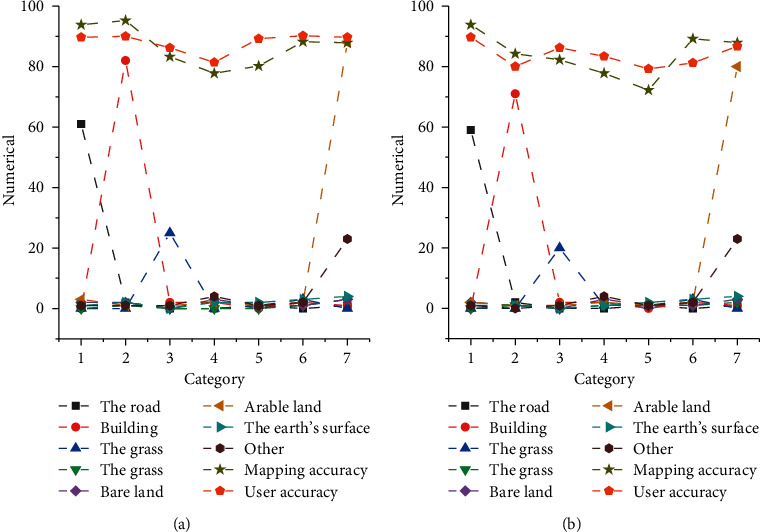
Results of the neural network classification and accuracy calculation (a) Results of the first test; (b) Results of the second test.

Figure 6 shows the classification results and the accuracy of the constructed CNN for the land information of the remote sensing image.

Figure 6 demonstrates that the constructed CNN can achieve 91.32% overall accuracy for black soil classification, 97.73% for rivers, canals, and lakes, and 93.63% for user accuracy. The proposed neural network method can be well applied to the extraction of land information from remote sensing images.

**Figure 7:**
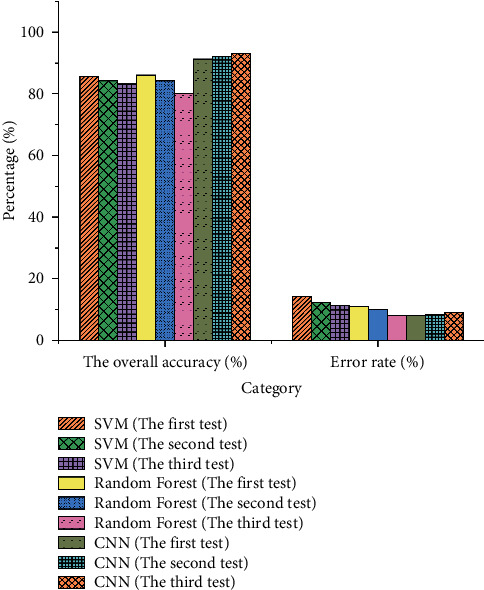
The result of calculating the accuracy of each classifier.

The result of calculating the accuracy of the classifier is illustrated in Figure 7.

Figure 7 indicates that in the first test of the SVM algorithm, the overall accuracy of the test is 85.66%, and the error rate is 14.32%. In the second test of SVM, the overall accuracy of the test is 84.22%, and the error rate is 12.22%. In the third test of SVM, the overall accuracy of the test is 83.22%, and the error rate is 11.37%. In the first test of the RF algorithm, the overall accuracy of the test is 86.12%, and the error rate is 10.85%. In the second test of the RF, the overall accuracy of the test is 84.22%, and the error rate is 9.89%. In the third test of RF, the overall accuracy of the test is 80.21%, and the error rate is 8.21%. In the first test of the CNN algorithm, the overall accuracy of the test is 91.31%, and the error rate is 8.21%. In the second test of the CNN, the overall accuracy of the test is 92.11%, and the error rate is 8.33%. In the third test of the CNN, the overall accuracy of the test is 93.22% and the error rate is 8.99%.

An evolutionary game model between the government and farmers is constructed using the method of the evolutionary game theory. Through the solution of the system evolutionary stability strategy and the analysis of the dynamic simulation results, it is found that: 1. From the influence of the government's choice of policy stimulus, it is necessary for the government to implement policies or their combination to stimulate farmers to adopt conservation tillage techniques, but the three policy incentives have different effects on farmers' adoption behavior. 2. Different from the conclusion that the subsidy policy has an incentive and effective promotion effect on the adoption of agricultural technology, if the government adopts the subsidy measures alone, the incentive effect of the farmers' adoption behavior is not good, that is to say, under the current government subsidies, without supporting other incentive tools, farmers will tend to choose traditional farming techniques in the final stable state. Therefore, subsidies need to be combined with punishment mechanisms or information-induced measures to play an effective role in promoting. 3. In terms of policy choice, compared with the effect of a single policy stimulus, a reasonable combination of the three policies has the best incentive effect on farmers' adoption of the conservation tillage technology, i.e., adding alternative tools under the same conditions, the effect of its combination can reach a stable state in which farmers tend to choose the conservation tillage technology in advance. 4. From the perspective of the characteristics of the policy combination and the dynamic simulation results, it is further found that with the passage of time, with appropriate regulatory measures in the long run, the government can gradually relax the stimulus of other policies, and farmers will gradually and spontaneously choose conservation tillage techniques.

## 6. Conclusion

Conservation tillage strategies are conducive to restoring cultivated land and protecting food security. However, various obstacles are encountered in the implementation of conservation tillage strategies. By analyzing the evolutionary game of different types of farmers and local governments' fallowing interests, conflicting focus, and behavioral strategies in the conservation tillage strategy pilot areas, the main problems are analyzed, and effective countermeasures and suggestions are provided to promote the smooth implementation of the fallow policy. However, there are still some shortcomings, which can be divided into the following two points: (1) The stakeholders in the implementation of the conservation tillage strategy are not only farmers and local governments, but also enterprises and the central government. Among them, there is a game relationship among the farmers, enterprises, local governments, and the central government. These game relationships can be investigated in future research. (2) The research subjects are mainly small-scale farmers. With the progress of land scale in China, farmers gradually change from small-scale farmers to large-scale farmers. Large-scale farmers can intensively plant crops and can achieve large-scale planting. Whether the country should implement fallow and the subsidy standard for fallow for large farmers also needs further study.

## Figures and Tables

**Table 1:** The game payoff matrix between the government and farmers.

Farmers	Government	
	Regulation	Not regulation
Adoption	−*C*_*f*_+*R*_*i*_+*R*_*e*_, −*C*_*g*_+*λR*_*i*_+*μR*_*e*_	−*C*_*f*_+*R*_*i*_+*R*_*e*_, −*βC*_*g*_+*λR*_*i*_+*μR*_*e*_
Not adoption	− *αC*_*f*_ − *F* − *αL*, −*C*_*g*_+*F* − *αμL*	−*αC*_*f*_*βF* − *αL*, −*αC*_*g*_+*βF* − *αμL*

**Table 2:** Results of the equilibrium point of Jacobi matrix det*J* and tr*J*.

Equilibrium point	Types	Results
(0, 0)	det*J*	[−*C*_*f*_(1 − *α*)+*βF*+*αL* − *R*_*i*_ − *R*_*e*_](*F* − *C*_*g*_)(1 − *β*)
	tr*J*	[−*C*_*f*_(1 − *α*)+*βF*+*αL* − *R*_*i*_ − *R*_*e*_]+0*v*(1 − *β*)
(0, 1)	det*J*	−[*F* − *C*_*f*_(1 − *α*)+*αL* − *R*_*i*_ − *R*_*e*_](*F* − *C*_*g*_)(1 − *β*)
	tr*J*	[*F* − *C*_*f*_(1 − *α*)+*αL* − *R*_*i*_ − *R*_*e*_] − (*F* − *C*_*g*_)(1 − *β*)
(1, 0)	det*J*	[−*C*_*f*_(1 − *α*)+*βF*+*αL* − *R*_*i*_ − *R*_*e*_]*C*_*g*_(1 − *β*)
	tr*J*	−[−*C*_*f*_(1 − *α*)+*βF*+*αL* − *R*_*i*_ − *R*_*e*_](−*C*_*g*_)(1 − *β*)
(1, 1)	det*J*	[*F* − *C*_*f*_(1 − *α*)+*αL* − *R*_*i*_ − *R*_*e*_](−*C*_*g*_)(1 − *β*)
	tr*J*	−[*F* − *C*_*f*_(1 − *α*)+*αL* − *R*_*i*_ − *R*_*e*_]+*C*_*g*_(1 − *β*)
(*x*^*∗*^, *y*^*∗*^)	det*J*	[*C*_*f*_(1 − *α*) − *βF* − *αL*+*R*_*i*_+*R*_*e*_](*F* − *C*_*g*_)(1 − *β*)/(1 − *β*)^2^(1 − *x*^*∗*^)(1 − *y*^*∗*^)
	tr*J*	0

**Table 3:** Stability of the equilibrium point.

Equilibrium point (*x, y*)	Types	Inference 1		Inference 2		Inference 3		Inference 4		Inference 5		Inference 6	
		Symbol	Stability	Symbol	Stability	Symbol	Stability	Symbol	Stability	Symbol	Stability	Symbol	Stability
(0, 0)	det*J*	+	Unstable	−	Saddle point	+	ESS	−	Saddle point	+	ESS	−	Saddle point
	tr*J*	+		Uncertain		−		Uncertain		−		Uncertain	
(0, 1)	det*J*	−	Saddle point	+	Unstable	−	Saddle point	+	ESS	+	Unstable	−	Saddle point
	tr*J*	Uncertain		+		Uncertain		−		+		Uncertain	
(1, 0)	det*J*	+	ESS	+	ESS	−	Saddle point	−	Saddle point	−	Saddle point	−	Saddle point
	tr*J*	−		−		Uncertain		Uncertain		Uncertain		Uncertain	
(1, 1)	det*J*	−	Saddle point	−	Saddle point	+	Unstable	+	Unstable	−	Saddle point	−	Saddle point
	tr*J*	Uncertain		Uncertain		+		+		Uncertain		Uncertain	
(*x*^*∗*^, *y*^*∗*^)	det*J*	Nonexistence		Nonexistence		Nonexistence		Nonexistence		Nonexistence		+	Center point
	tr*J*											0	

## Data Availability

All data used to support the findings of the study can be obtained from the corresponding author upon request.
